# RNA-seq analysis of the hypothalamic transcriptome reveals the networks regulating physiopathological progress in the diabetic GK rat

**DOI:** 10.1038/srep34138

**Published:** 2016-09-28

**Authors:** Yuhuan Meng, Yujia Guan, Wenlu Zhang, Yu-e Wu, Huanhuan Jia, Yu Zhang, Xiuqing Zhang, Hongli Du, Xiaoning Wang

**Affiliations:** 1 School of Bioscience & Bioengineering, South China University of Technology, Guangzhou, 510006, China; 2 BGI-Shenzhen, Shenzhen, 518031, China; 3 Guangdong Key Laboratory of Laboratory Animals, Guangzhou, 510663, China; 4 Chinese PLA General Hospital, Beijing, 100853, China

## Abstract

The Goto-Kakizaki (GK) rat is an animal model of non-obese type 2 diabetes (T2D). The GK rat was generated through the introduction of various genetic mutations from continuous inbreeding; these rats develop diabetes spontaneously. The mutated genes in GK rats may play key roles in the regulation of diabetes. The hypothalamus plays a central role in systematic energy homeostasis. Here, the hypothalamic transcriptomes in GK and Wistar rats at 4, 8 and 12 weeks were investigated by RNA-seq, and multiple variants and gene expression profiles were obtained. The number of variants identified from GK rats was significantly greater than that of Wistar rats, indicating that many variants were fixed and heritable in GK rats after selective inbreeding. The differential gene expression analysis indicated that GK rats had a dysfunctional hypothalamic melanocortin system and attenuation of the hypothalamic glucose-sensing pathway. In addition, we generated integrated gene network modules by combining the protein-protein interaction (PPI) network, co-expression network and mutations in GK and Wistar rats. In the modules, GK-specific genes, such as *Bad*, *Map2k2, Adcy3, Adcy2* and *Gstm6*, may play key roles in hypothalamic regulation in GK rats. Our research provides a comprehensive map of the abnormalities in the GK rat hypothalamus, which reveals the new mechanisms of pathogenesis of T2D.

Type 2 diabetes (T2D), which is characterized by hyperglycaemia, insulin resistance of target tissues and insufficient insulin secretion from pancreatic β-cells, is a worldwide epidemic that affects more than 415 million people^1^. Approximately one in four cases of diabetes worldwide occur in China. Moreover, T2D develops at considerably lower BMI values in the Chinese population compared with that of Western white populations^2^,^3^.

The Goto-Kakizaki (GK) rat is one of the best characterized animal models of non-obese T2D and displays hyperglycaemia, β-cell defects and insulin resistance. GK rats spontaneously develop diabetes and were generated through repeated inbreeding of Wistar rats selected at the upper limit of the normal distribution for glucose tolerance^4^. The pathogenesis of the GK rat may result from the loss of β-cells and impairment of β-cell function, which depends on various genetic factors and certain trans-generational epigenetic stimuli^5^,^6^,^7^. The susceptibility loci containing genes responsible for several diabetic traits may play fundamental roles in the development of T2D in GK rats^7^. GK rat embryos transplanted into Wistar rats still developed diabetes, indicating that genetic factors play much more important roles in pathogenesis than the gestational environment^8^. In addition to hyperglycaemia, β-cell defects and insulin resistance, GK rats also exhibited hyperphagia, but the average body weight was 10–30% less than that of Wistar rats^6^,^9^,^10^,^11^.

The hypothalamus controls food intake and plays a central role in energy homeostasis^12^. Additionally, the hypothalamus influences peripheral organs to control the degree of energy expenditure and feeding behaviour by sensing hormones, such as insulin, leptin and ghrelin, released from peripheral tissues, and nutrients, such as glucose, amino acids and fatty acids, from the circulatory system^13^,^14^. A previous study reported that increased *Npy* mRNA levels in the hypothalamus led to hyperphagia in the GK rats^11^, but the regulatory network in the GK rat hypothalamus is unclear. Moreover, the hypothalamus plays an important role in obesity and can increase the risk for diabetes^15^,^16^,^17^. In addition, dysfunction of the hypothalamus may result in a series of metabolic disorders, and the role of the hypothalamus in the non-obese GK rat model is unclear.

To investigate the role of the hypothalamus in T2D pathogenesis in GK rats, we analysed the transcriptomes of the GK rat hypothalamus at 4, 8 and 12 weeks by RNA-seq. The fixed mutations and differentially expressed genes were analysed. By combining the protein-protein interactions (PPI) network, co-expression network and mutations, we generated integrated network modules enriched for GK mutation genes. These modules can help elucidate gene regulation of the hypothalamus and the roles of the hypothalamus in the pathogenesis of diabetes in GK rats.

## Results

### Variants in GK and Wistar rat hypothalami

**Figure 1:**
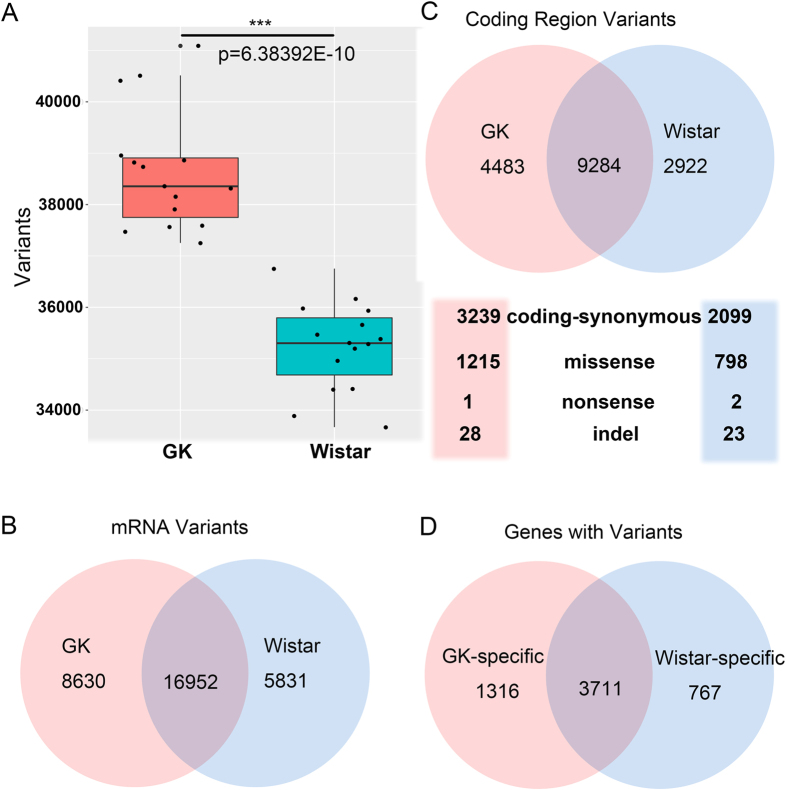
Variants in GK and Wistar rats. (**A**) The number of variants from GK (orange) and Wistar rats (cyan). (**B**) Venn diagram representing the overlap of common variants between GK and Wistar rats in the mRNA regions. (**C**) The common variants between GK and Wistar rats in the coding regions and the details of GK-specific and Wistar-specific mutations are listed. (**D**) Genes with variants in GK and Wistar rats.

The variants in GK rats play key roles in the development of T2D because the diabetic GK rats were established by repeated inbreeding of Wistar rats with glucose intolerance as a selection index^4^. Here, the variants in GK and Wistar rats were determined by mapping their reads to the verified reference mRNA sequences of *Rattus norvegicus* (Norway rat). The number of variants from GK rats was significantly greater than those of Wistar rats (p < 0.001, Student’s t test; Fig. 1A), indicating that many variants were fixed and heritable in GK rats after selective inbreeding.

We found 22,783 and 25,582 common variants in 15 Wistar rats and 15 GK rats, respectively. Overall, 31,380 variants were detected in hypothalamic transcripts. Among them, 16,952 overlapping variants were found in both Wistar and GK rats. There were 5831 and 8630 specific variants in Wistar and GK rats, respectively (Fig. 1B). Among all variants, we obtained 16,685 variants in the coding regions, and 9284 overlapping variants were found in both Wistar and GK rat samples. GK rats had 4483 specific variants, including 3239 synonymous mutations, 1215 missense mutations, 28 indels and 1 nonsense mutation. Wistar rats had 2922 specific variants, comprising 2099 synonymous mutations, 798 missense mutations, 23 indels and 2 nonsense mutations (Fig. 1C). After the analysis, 1316 genes with variants were detected only in GK rats, which were defined as GK-specific genes; whereas the number of Wistar-specific genes was 767 (Fig. 1D). Among the GK-specific genes, 488 genes had at least one missense or indel mutation, and these genes were more likely to influence the development of T2D in the GK rats, at least at the amino acid level. Detailed information on the variants and corresponding genes is listed in Table S1.

### Differentially expressed genes and their relationship with the hypothalamic melanocortin system and glucose-sensing pathway

To assess the impact of hypothalamic regulation on T2D development in GK rats, the differentially expressed genes of the hypothalamus at 4, 8 and 12 weeks were obtained by bioinformatics analyses. In total, there were approximately 13,600 genes detected with expression in the hypothalamus [mean FPKM(fragments per kilobase of exon per million fragments mapped) > 1]. Specifically, 13,626, 13,698 and 13,654 genes were detected at 4, 8 and 12 weeks, respectively. Compared with Wistar rats, 1119, 1381 and 1702 differentially expressed genes in GK rats were found at 4, 8 and 12 weeks, respectively (with p < 0.05, FDR correction). The comparison of gene expression between Wistar and GK rats is shown in Table S2.

#### Hypothalamic melanocortin system

**Figure 2:**
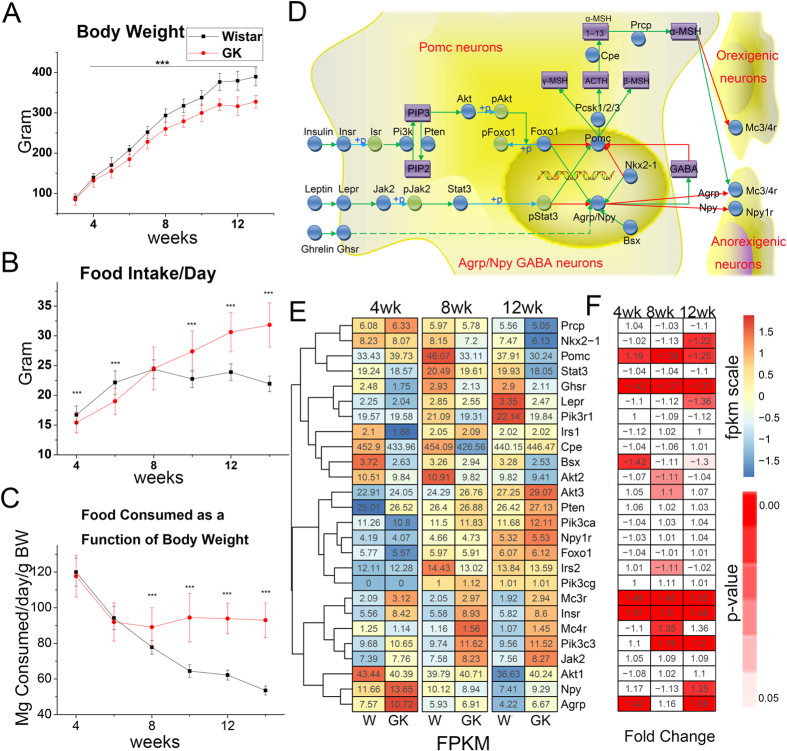
Physiological index and the hypothalamic melanocortin system. (**A**) Body weights (grams) of GK and Wistar rats. (**B**) Daily food intake (grams) of GK and Wistar rats. (**C**) Daily food consumption (mg) adjusted for body weight (grams). Data are shown as the mean ± SD. Black = Wistar, red = GK. ***p < 0.01. (**D**) Hypothalamic melanocortin pathways. The Agrp/Npy neurons express ghrelin receptors, which can specifically detect ghrelin, and positively regulate the expression of *Agrp/Npy*. Agrp/Nny neurons also express leptin and insulin receptors, which detect leptin and insulin, respectively, and negatively regulate the AgRP/NPY neuronal activity. The Agrp/Nny neurons release *Agrp* and *Npy*, negatively regulate the anorexigenic system and increase the food intake. By contrast, subtypes of Pomc neurons produce either leptin or insulin receptors that sense adipose-derived leptin or pancreas-derived insulin, express *Pomc* and then produce α-MSH by enzymatic processing. Finally, α-MSH positively responds to the anorexigenic molecules and negatively responds to orexigenic neurons and further decreases the food intake. In addition, γ-amino-butyric acid (GABA) released by Agrp/Npy neurons can inhibit the activity of Pomc neurons. (**E**) The gene expression pattern in hypothalamic melanocortin system. (**F**) The fold change and the corresponding p-value of each gene in GK compared with Wistar rats.

The physiological data showed that the body weights of GK rats were significant lower than those of Wistar rats from 4 weeks onwards (Fig. 2A), and the average body weights of GK rats were less than those of Wistar rats, from 5.4% (4 weeks) to 16.7% (12 weeks), which was similar to previous GK rat studies^9^,^10^. Additionally, the food intake of GK rats was significant higher than that of Wistar rats after 10 weeks (Fig. 2B). After adjusting for body weight, the actual food intake of GK rats was similar to that of Wistar rats at 4 and 6 weeks, but significantly higher than those of Wistar rats from 8 weeks to 12 weeks (Fig. 2C). The abnormality in food consumption from 8 weeks on may have a strong correlation with metabolic disorders in GK rats. In the hypothalamus, the melanocortin system controls energy homeostasis and feeding behaviour^12^ (Fig. 2D). In our data, we found many differentially expressed genes in the melanocortin system, and the detailed mean FPKM and p-values of these genes are shown in Fig. 2E,F. Among them, *Pomc* (proopiomelanocortin) in the GK rat hypothalamus was significantly lower than that of the Wistar rat hypothalamus at 8 weeks (p = 0.0026, DFR correction), which may result in the hyperphagia observed in GK rats. *Agrp* (agouti-related neuropeptide) and *Npy* (pro-neuropeptide Y preproprotein) in the GK rat hypothalamus were significantly higher than those of Wistar rats in the hypothalamus at 12 weeks (*Agrp* p = 0.017, FDR correction; *Npy* p = 0.012), which may result in the increased food intake in GK rats. In addition, the *Agrp* and *Npy* expression changed over time. Compared with the expression at 4 weeks, *Agrp* and *Npy* were both decreased at 8 and 12 weeks (p < 0.05, two-way ANOVA test, Table S2), and relative food intake showed the same trend as *Agrp* and *Npy* (Fig. 2C), which indicated a positive correlation between *Agrp/Npy* and food intake after adjusting for body weight.

#### Hypothalamic glucose-sensing pathway

**Figure 3:**
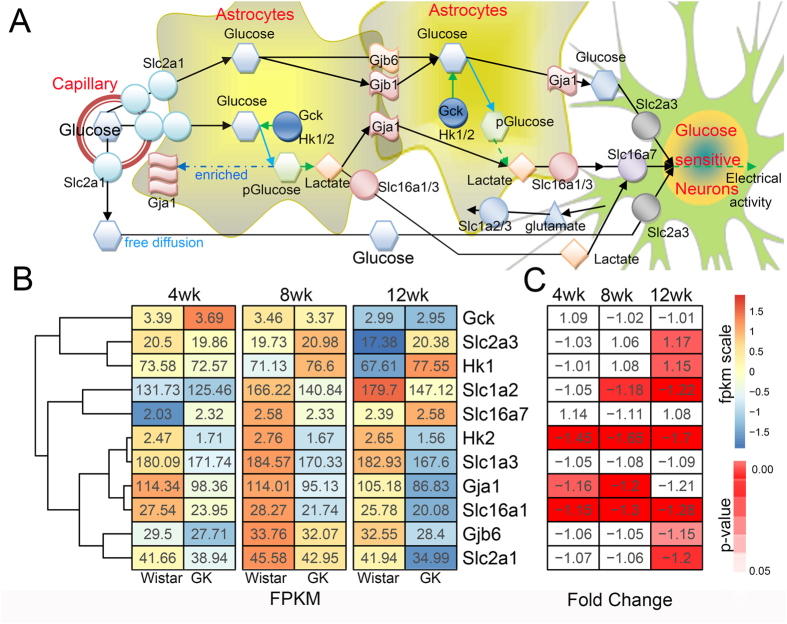
Glucose sensing in the hypothalamus. (**A**) The glucose-sensing pathway in the capillary-astrocytes-neurons axis. Blood glucose in the vessels reaches the hypothalamus through endothelial *Slc2a1* (also known as *Glut1*). *Slc2a1*, also expressed in astrocytic endfeet around the blood vessels, can detect and transport glucose into the astrocytes. After transport into the cells, the glucose is quickly phosphorylated by the hexokinase (*Hk1/2*) or glucokinase (*Gck*) in the astrocytes. The phosphorylation of glucose then results in enrichment of *Gja1* (known as *Cx43*) in the astroglial endfeet wrapping the blood vessels. Lactate, which is metabolized by glucose in a series of enzymatic steps, can cross into the astrocytes by both *Gja1* and *Gjb6* (*Cx30*) gap-junctions or alternatively can be transported to the extracellular space with the help of astroglial *Slc161*/*3* (*Mct1/3*). Finally, the extracellular lactate was transported through *Slc16a7(Mct2)* into the glucose-sensitive neurons (GSN). The oxidation of lactate further regulates the GSN electrical activity. Additionally, *Slc2a3 (Glut3)*, which was expressed in the GSN, can detect and transport glucose released from the astrocytes through *Gja1* or the freely diffusing glucose at the endothelial site. Upon neuronal activation, the glutamate release from the GSN will be detected and transported into the astrocytes by astroglial *Slc1a2* or *Slc1a3*, and it will further influence the expression of *Gja1* in the astrocytes. (**B**) The gene expression in the hypothalamic glucose-sensing pathway. (**C**) The fold change and the corresponding p-value of each gene in GK rats compared with Wistar rats.

Astrocytes are necessary for hypothalamic glucose sensing, which is part of the regulation of energy homeostasis^13^,^14^ (Fig. 3A). In our study, several genes involved in glucose sensing showed differences between GK and Wistar rats. The FPKM, fold change and corresponding p-value of the glucose sensing genes are shown in Fig. 3B,C. *Slc16a1* and *Slc6a3*, also known as *Mct1* and *Mct3*, respectively, can transport lactate, the major molecule of glucose sensing in neurons, from astrocytes to the extracellular space. In our data, the expression of *Slc6a3* was very low (mean FPKM < 1), whereas *Slc16a1* was down-regulated at all periods in GK rats (p < 0.01). The gap-junction genes *Gja1* and *Gjb6* (*Cx43* and *Cx30*), which participate in transporting glucose and lactate between astrocytes and neurons, were differentially expressed between GK and Wistar rats. *Gja1* was lower in the GK rat hypothalamus at all stages, with down-regulation at 4 and 8 weeks (p = 0.019 and p = 0.0078 with FDR correction), whereas *Gjb6* was down-regulated only at 12 weeks (p = 0.028). *Slc1a2* and *Slc1a3* (*Glt1* and *Glast*), which promote glutamate release from glucose-sensitive neurons (GSN) into astrocytes, were detected. The expression of *Slc1a2* was decreased in the GK rat hypothalamus at 8 and 12 weeks (p = 0.013 and p = 0.015 with FDR correction), whereas *Slc1a3* exhibited no difference between GK and Wistar rats. *Slc2a1* (also known as *Glut1*), which was found in endothelial cells of the central nervous system (CNS) blood vessels and astrocytes in rat brain^18^ and is a carrier that transports glucose from the blood to astrocytes, was decreased at 12 weeks (p = 0.01 with FDR correction). The genes described above were expressed in astrocytes, and their down-regulation in GK rats would weaken the transfer of glucose, lactate or glutamate in astrocytes, which indicated that glucose sensing in the GK rat hypothalamus might be impaired. Additionally, the impairment of glucose sensing is likely to disrupt energy homeostasis in the hypothalamus and peripheral organs.

### Integrated gene networks in the GK rat hypothalamus

**Figure 4:**
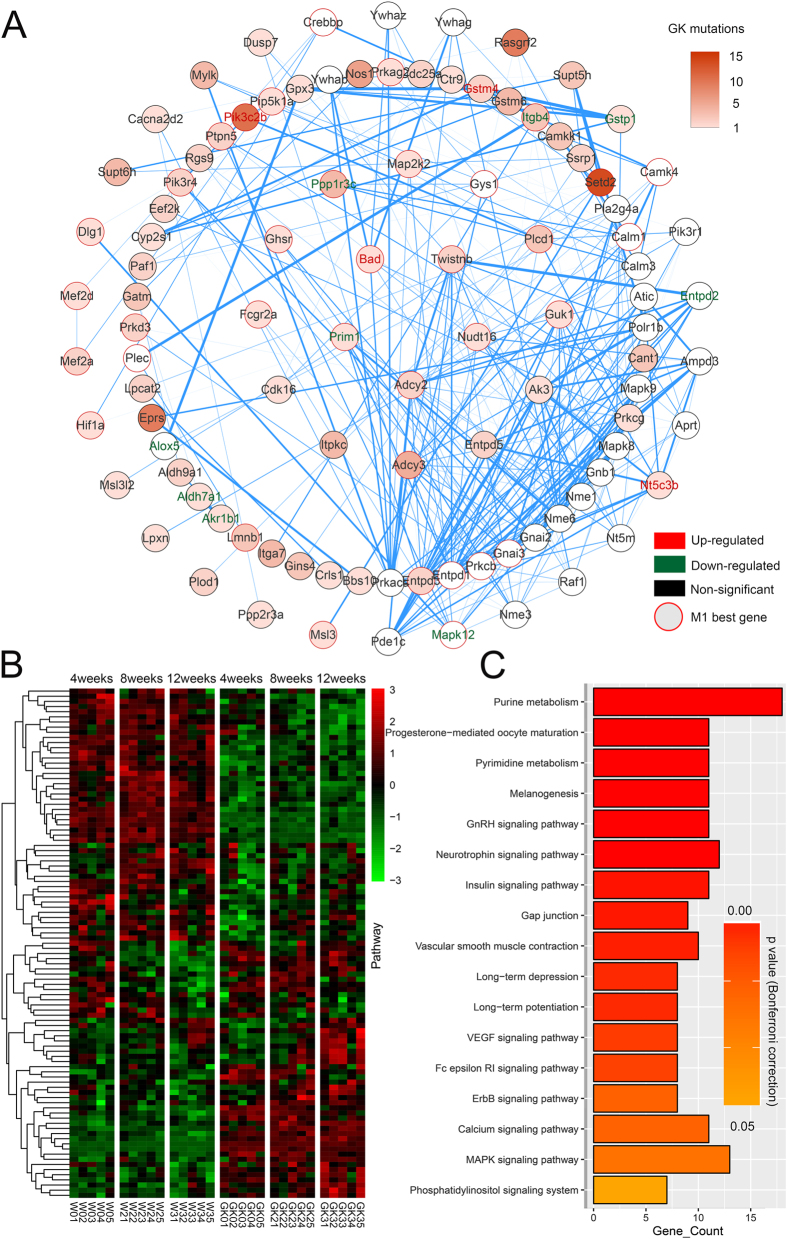
Module M1. (**A**) The gene network in M1_Extended. Specifically, node colours reflect the number of mutations in GK rats: the more intense orange colour indicates a higher number of mutations, whereas white indicates no mutation in GK rats. Edges (blue lines) between two nodes represent genes that interact with each other according to the PPI network, and the weight of the edges represents the coexpression coefficient r2. The genes in the innermost circle were detected in >99% of the suboptimal modules. Genes in subsequent concentric circles were found in > 80%, 20%, and 5% (M1_Extended) of the suboptimal modules, respectively. Nodes with red outlines represent the genes belonging to M1_best. The gene names in the nodes that are up-related and down-related in GK rats compared with Wistar rats are denoted in red and green, respectively. (**B**) The heatmap of the expression of M1_Extended genes (FPKM) in each individual. (**C**) The significant KEGG pathway enrichment in M1_Extended, the gene counts and the p-value corrected by Bonferroni are shown.

**Figure 5:**
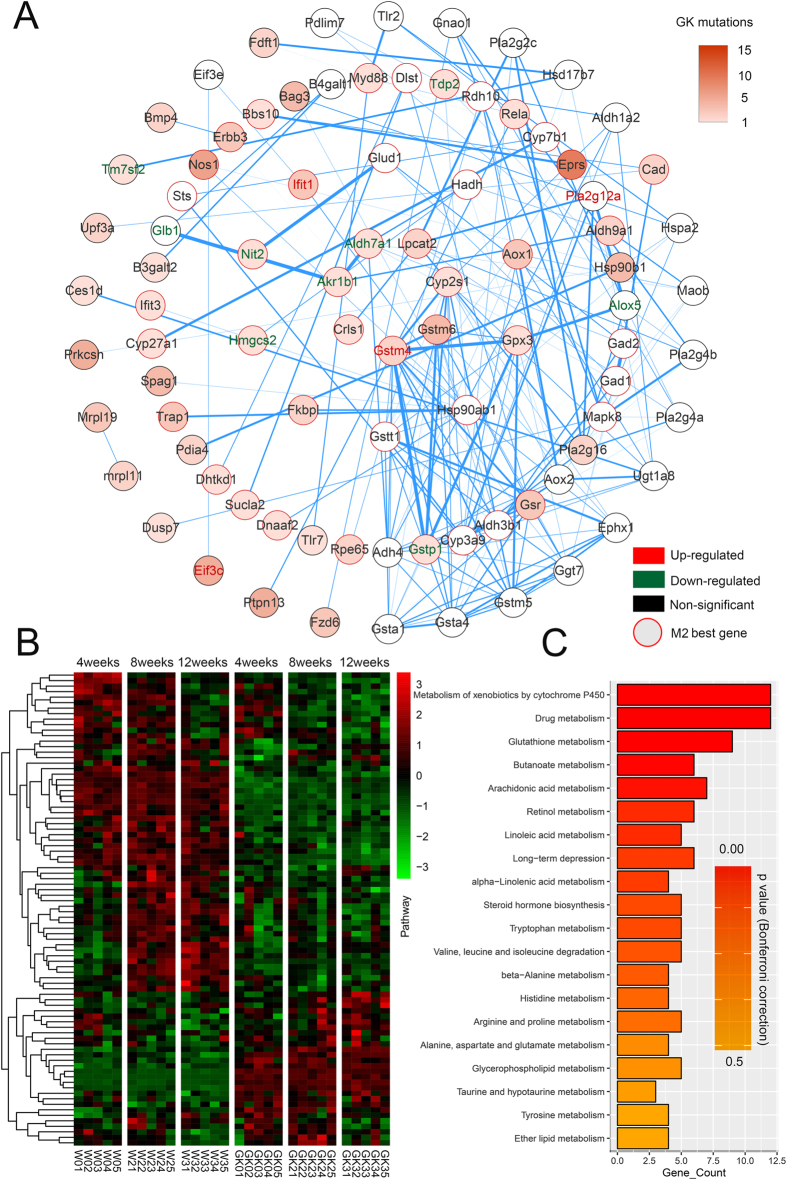
Module M2. (**A**) The gene network in *M2_Extended*. (**B**) The heatmap of expression of the *M2_Extended* genes (FPKM) in each individual. (**C**) The pathway enrichment in KEGG pathways in *M2_Extended*. Gene counts and Bonferroni corrected p-values are shown.

Single omics data alone could not completely elucidate the disease mechanism. Therefore it is essential to integrate omics data at different levels to efficiently identify new mechanisms. Here, we used the PPI, and co-expression networks of the transcriptomes at 4, 8 and 12 weeks, together with mutations in GK and Wistar rats to generate highly connected modules that were enriched for gene mutations in GK rats using MAGI(merging affected genes into integrated networks)^19^. The results of MAGI PathwaySelect showed that the seed pathway with the highest score, including eight genes, *Gpx3, Gstm6, Cyp2s1, Gstm4, Aldh3b2, Carns1, Aldh9a1* and *Akr1b1*, was significantly enriched in the KEGG pathways glutathione metabolism (p = 1.6E-2, Bonferroni correction) and metabolism of xenobiotics by cytochrome P450 (p = 2.3E-2, Bonferroni correction) and the GO annotation oxidation reduction (p = 4.5E-2, Bonferroni correction). In addition, among all the local optimal solutions in MAGI, we defined the module with the highest score as the Best Module (*M_Best*) and defined genes that appeared in >5% of the suboptimal modules as *M_Extended*. Eventually, by combining the seed pathways and reiterating module discovery, we identified two distinct disjoint modules (M1 and M2), which may be associated with diabetogenic progression in GK rats (Figs 4 and 5, respectively).

#### Module 1

For module 1 (Fig. 4A), *M1_best* contains 49 genes, of which 36 genes were found in *M1_Extended*. *M1_Extended* contains 99 genes with 164 coding-synonymous and 70 missense mutations in GK rats. Among *M1_Extended* genes, 4 genes, *Bad* (1 missense), *Gstm4* (2 missense), *Pik3c2b* (8 synonymous and 2 missense) and *Nt5c3b* (1 synonymous), were significantly up-regulated in GK rats compared with Wistar rats at all periods from 4 to 12 weeks. Nine genes were significantly down-regulated including *Entpd2*, *Itgb4*(2 synonymous and a missense), *Ppp1r3c*(3 synonymous and 1 missense), *Aldh7a1*(1 missense), *Gstp1*(1 synonymous), *Mapk12*, *Alox5*, *Akr1b1*(1 missense) and *Prim1*(1 missense) (Table 1). The FPKM of all genes in M1 from all individuals is shown in a heatmap in Fig. 4B. There were 17 pathways strongly associated with *M1_Extended* (p < 0.05, Bonferroni correction, Fig. 4C), and 9 of them were also significantly enriched in *M1_best*. These pathways were GnRH, VEGF, neurotrophin, insulin signaling pathways, melanogenesis, long-term potentiation, phosphatidylinositol signaling system, gap junction and purine metabolism. It is well known that melanogenesis, progesterone-mediated oocyte maturation, GnRH and the insulin signaling pathway belong to the endocrine system, which is closely related to diabetes. The neurotrophin signaling pathway and long-term potentiation/depression belong to the nervous system, which may participate in the regulation of peripheral tissues by the hypothalamus. The signal transduction contains ErbB, calcium, the MAPK signaling pathway and the phosphatidylinositol signaling system, which are very important to control and maintain a normal physiological balance in the body. Every abnormal change in signal transduction could influence the organismal operation. Gap junctions, the VEGF signaling pathway and vascular smooth muscle contraction likely contribute to hypothalamic glucose sensing^14^.

#### Module 2

After excluding mutations and genes in *M1_Best*, we reran MAGI and discovered a second module, M2 (Fig. 5A,B). In module 2, *M2_best* contained 48 genes, of which 45 genes were found in M2_Extended. *M2_Extended* contained 86 genes comprising a total of 113 SNPs in GK rats (55 synonymous and 58 missense mutations). Among *M2_Extended* genes, four genes, *Gstm4* (2 missense), *Ifit1* (1 synonymous and 2 missense), *Pla2g12a* and *Eif3c* (4 synonymous and 1 missense), were significantly up-regulated in GK rats; nine genes were significantly down-regulated: *Aldh7a1* (1 missense), *Gstp1* (1 synonymous), *Tdp2* (1 missense), *Alox5*, *Glb1*, *Tm7sf2* (1 missense), *Akr1b1* (1 missense), *Nit2* (1 missense) and *Hmgcs2* (1 missense) (Table 1). There were 18 pathways detected in the KEGG pathway with p-values < 0.05, and most of them belong to metabolism (Fig. 5C). Among them, 7 pathways were significantly enriched with p < 0.05 after Bonferroni correction, including metabolism of xenobiotics by cytochrome P450, drug, glutathione, butanoate, arachidonic acid, retinol and linoleic acid metabolism (sorted by p value), and the top 4 pathways are overlapped in *M2_best*. The genes in M1 and M2 were enriched for GK rat mutations and high co-expression in the GK rat hypothalamus. The pathways enriched in these genes may play crucial roles in the regulation of the GK rat hypothalamus.

#### Sub modules of time course

**Figure 6:**
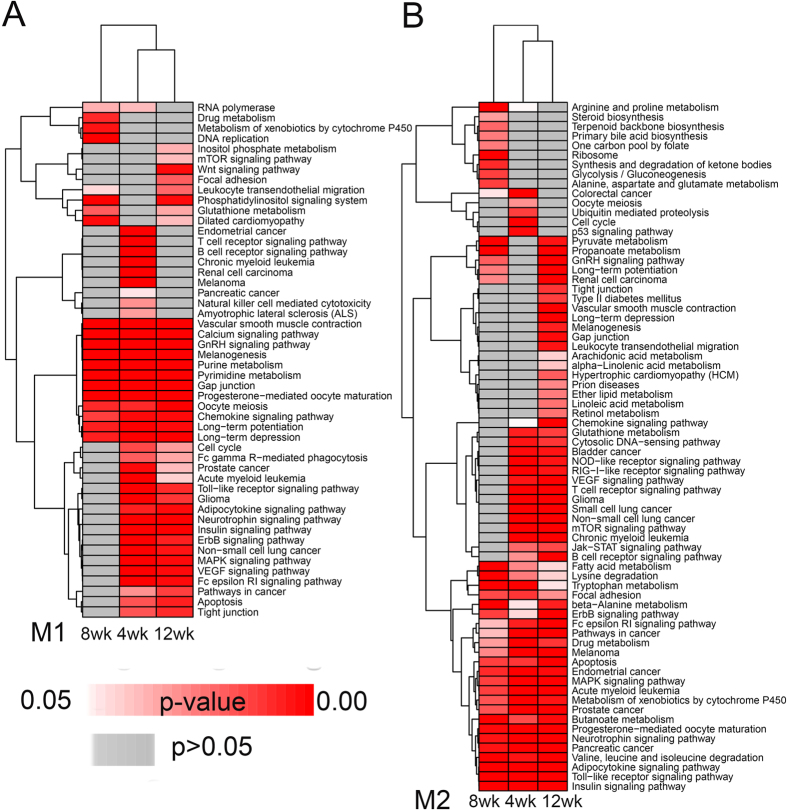
Pathways enriched in M1 and M2 by time courses. The significant KEGG pathway clustering in sub modules M1 (**A**) and M2 (**B**) at 4, 8 and 12 weeks.

To reveal the biological changes from 4 to 12 weeks in the hypothalamus and its contributions to diabetes development in GK rats, we reran MAGI at each stage of 4, 8 and 12 weeks and generated two significant sub modules (M1 and M2) for 4, 8 and 12 weeks (Table 1 and Table S3). Detailed information on the modules is presented in Table S3. After the KEGG pathway enrichment analysis of sub M1 and M2 in each period, we observed the pathways clustering as shown in Fig. 6. In sub module 1, the common pathways in all periods were similar to M1 pathway enrichment (related to the endocrine system, nervous system and signal transduction pathways) (Fig. 6A). The common pathways in sub module 2 were also similar to M2 pathways (enrichment in metabolism-related pathways), but pathways enriched in sub module 2 were more complex (Fig. 6B). The pathways that were significantly enriched in all stages may be important for T2D development in GK rats. However, some pathways appeared in only 1 or 2 periods, and the differences in the pathway enrichment at each stage may due to the alterations of *in vivo* environments, such as chronic hyperglycaemia (Supplemental Material Figure S1A), β-cell mass loss, dysfunction-induced decrease in insulin secretion (Supplemental Material Figure S1B), peripheral organism inflammation, insulin resistance and oxidative stress^7^. In general, the biological progresses were similar between 4 and 12 weeks but differed at 8 weeks. This phenomenon could be explained by a dynamic process of dysfunctions (4 and 12 weeks) and compensations (8 weeks) in diabetes development in GK rats. This explanation was also suggested in a previous GK rat liver network analysis^20^.

## Discussion

This study is the first to report the hypothalamic transcriptome and population variations in GK rats. Modules with integrated PPI, transcriptomic profile and GK rat mutation data were constructed to comprehensively elucidate hypothalamic regulation in diabetic progression in GK rats.

The causes of diabetes development in GK rats are hyperglycaemia, impaired insulin secretion, overproduction of hepatic glucose, and peripheral insulin resistance^9^. However, the fundamental cause, which may be due to the fixed variants associated with glucose intolerance, came from repeating breeding of glucose intolerant Wistar rats^4^. Several genes were detected with missense or indel mutations only in GK rats, such as Bcl2-antagonist of cell death (*Bad*), which contained a G > T variant at position 276 in the coding region (a missense mutation of Gln92His). *Bad* has been reported to have dual functions in apoptosis and glucose metabolism^21^,^22^ and could affect the glycolysis and gluconeogenesis in liver^23^ and the metabolism of glucose in the brain^24^. Growth hormone secretagogue receptor (*Ghsr*), which had a variant of T > A in the coding region and a corresponding missense mutation of V153E, is the receptor of ghrelin. Ghrelin has been shown to be an orexigenic peptide that antagonizes leptin action^25^. Other genes, such as *Agt*^26^, *Inppl1*^27^, *Ide*^28^ and *Sucla2*^29^, were related to insulin functions or glucose metabolism and had missense mutations in GK rats. These genes might contribute to the development of T2D. However, the variants were obtained only in the known transcripts in the hypothalamus. The variants in some tissue-specific genes, novel genes or imprinted genes were beyond the scope of our study. Moreover, the effects of variants in the transcription factor binding regions were also undetected in our study.

The genes of the melanocortin system in the hypothalamus regulate central energy metabolism. For example, *Pomc* could up-regulate anorexigenic neurons and down-regulate orexigenic neurons in the hypothalamus, and thus reduces food intake and increases energy expenditure, whereas *Agrp/Npy* could negatively regulate anorexigenic neurons and increase food intake and reduce energy expenditure^12^. In our study, the significant down-regulation of *Pomc* at 8 weeks and the significant up-regulation of *Agrp*/*Npy* at 12 weeks could partly explain the hyperphagia observed in GK rats from 8 to 12 weeks (Fig. 2 and Supplemental Material Figure S2A). *Npy* increases in GK rats also have been reported at 11 weeks^11^. Neuron-specific disruption of the insulin receptor (*Insr*) or decreasing hypothalamic *Insr* would cause hyperphagia^30^,^31^. The *Insr* positively regulates the expression of *Pomc* and negatively regulates *Agrp/Npy* via the insulin signaling pathway in the hypothalamus^12^. Unlike *Insr*, the growth hormone secretagogue receptor (*Ghsr*) positively regulates the expression of *Agrp/Npy* and negatively regulates *Pomc*^12^,^32^. NK2 homeobox 1 (*Nkx2-1)* activates *Agrp* gene expression but inhibits *Pomc* transcriptional activity by binding to the corresponding cis-acting elements in *Agrp* and *Pomc* gene promoters^33^,^34^. The brain-specific homeobox transcription factor (*Bsx*) can trigger *Agrp* but not *Pomc* gene expression^35^. The up-regulation of *Insr* and down-regulation of *Ghsr*, *Nkx2-1* and *Bsx* in our data (Fig. 2E,F) should result in the down-regulation of *Agrp/Npy* and up-regulation of *Pomc*. However *Agrp* and *Npy* were increased and *Pomc* was decreased in the present study (Fig. 2E,F), which was inconsistent with the previous studies. Dysfunction in the GK rat hypothalamic melanocortin system was found in the present study, which resulted in hyperphagia in GK rats and further disrupted energy homeostasis.

The astrocytes in the hypothalamus can transport glucose or lactate into the GSN, and then the GSN will initiate adaptive responses to control hepatic glucose production^14^. K_ATP_ channels are widely distributed in the hypothalamus and have been implicated in brain glucose sensing^13^,^36^. Detection of glucose in the hypothalamus can regulate the hepatic glucose production via K_ATP_ channels^37^,^38^. In GK rats, the hepatic glucose production was increased^39^. Our results indicated that glucose detection in the astrocytes may be impaired in the hypothalamus. The expression of glucose-sensing genes in astrocytes, such as the transport genes (*Slc1a2, Slc2a1* and *Slc16a1*) and gap junction genes (*Gja1* and *Gjb6*), was decreased (Fig. 3 and Supplemental Material Figure S3B), which may weaken the transport of glucose and lactate to glucose-sensitive neurons. The impairment of the hypothalamic glucose-sensing pathway in GK rats may further influence hepatic glucose production.

*Bad* was present in the *M1_best* and all M1 sub modules (Table 1 and Fig. 4A). Bad is the upstream signal of the K_ATP_ channel in the brain that controls neuronal excitation^24^. The phosphorylation of serine 155 at the Bad BH3 domain can increase glucose and decrease K_ATP_ channel activity^24^. Closure of the K_ATP_ channel results in increased GABA in the hypothalamus^40^ and also affects the expression of *Pomc* and food intake. Bad was significantly up-regulated in the GK rat hypothalamus (Table S2 and Supplemental Material Figure S2C), which may indirectly decrease *Pomc* (Fig. 2E,F) and increase food intake (Fig. 2C) in GK rats. The missense mutation in Bad in GK rats (G276T) was not in the BH3 region (positions 442 to 486); thus further study is needed to determine whether this missense mutation influences the phosphorylation of serine 155.

There were 11 genes (*Raf1, Map2k2, Prkcg, Prkcb, Prkaca, Mapk9, Mapk8, Calm3, Calm1, Adcy3* and *Adcy2*) that appeared at least 7 times in 17 M1 significant pathways (Table S3). Among them, *Map2k2* (1 missense), *Adcy3* (4 synonymous and 1 missense) and *Adcy2* (1 synonymous and 1 missense) were GK-specific genes that had mutations only in GK rat samples. The mutation G876A in *Map2k2* is a dbSNP (id: 8158310), which results in an amino acid change from Val to Met. This mutation is in the catalytic domain of the dual-specificity protein kinase and may affect the catalysis of mitogen-activated protein (MAP) or extracellular signal-regulated kinase (ERK) kinase. *Map2k2* is involved in many signal transduction pathways and contributes to intracellular signaling cascades. *Map2k2* is also involved in the insulin signaling pathway and has been correlated with metabolic traits, such as body mass index, fasting glucose, and fasting insulin, which may regulate steroidogenesis and glucose homeostasis^41^. *Adcy3* and *Adcy2* are both adenylyl cyclases that catalyse the synthesis of 3′-5′-cyclic adenosine monophosphate (cAMP). Genetic polymorphisms in Adcy3 were associated with obesity^42^,^43^. In addition, loss of *Adcy3* in the mouse hypothalamus can lead to obesity^44^. These studies indicated that *Adcy3* plays an important role in the regulation of body weight^45^. In our study, Adcy3 had 4 mutations. Among them, T803C was a missense causing a Leu to Pro change in the Adcy3 transmembrane region (from 754 to 816). *Adcy3* was increased in the GK rat hypothalamus at 10 weeks^46^. According to our data, *Adcy3* had the same results at 12 weeks (p = 0.015) but not at 4 and 8 weeks, which indicated that *Adcy3* might play a key role in preventing obesity in GK rats despite the hyperphagia from 8 weeks on. Nine genes, including *Cyp3a9, Ugt1a8, Gstt1, Gstp1, Gstm6, Gstm5, Gstm4, Gsta4* and *Adh,* appeared at least 3 times in 7 M2 significant pathways (Table S3). Only *Gstm6*(3 synonymous and 1 missense mutations in GK rats) was a GK-specific gene. The four mutations in *Gstm6* were in the provisional glutathione S-transferase region (positions 118 to 717), including the missense mutation G319A (Val > Ile), and may affect the polypeptide binding. *Gstm6* was down-regulated in GK rats at all stages and is related to the metabolism of xenobiotics by cytochrome P450, drug metabolism and glutathione metabolism pathways. *Gstm6* was reduced in both diabetic and insulin resistant mice and may be involved in the progression of the syndrome from insulin resistance to type 2-like diabetes^47^. Although comprehensive modules and mechanisms in the GK rat hypothalamus were obtained by integrating PPI, transcriptomic profiles and mutations in GK rats, one limitation of the method is that the current protein interaction network databases are largely incomplete, and not all biological interactions involved have been annotated.

High-fat-diet-induced rats showed inflammation in the hypothalamus on the first day^17^, which is critical in development of obesity T2D^15^,^16^. Among the pathways in M1 and M2, only the Fc epsilon RI signaling pathway was related to the immune system. The expression of pro-inflammatory factors, such as *Il-1b*, *Tnf* and *Il-6*, were low (FPKM < 1) and were not significantly different between GK and Wistar rats, as verified by qPCR (Supplemental Material Figure S3), which indicated that there was no inflammation in the GK rat hypothalamus at the early stage. The hypothalamic regulation of GK rats and high-fat diet rats appears to be different. Additionally, the expression of *Bad* in ob/ob mice showed no significant differences compared with the controls in the hypothalamus (data from GEO datasets: GSE10785^48^, GSE61436 and GSE62013^49^), whereas *Bad* expression was much higher in GK rats than in Wistar rats. *Bad* knockout mice have reduced glucose and increased metabolism of ketone bodies in brain cells, which produced a marked increase in the activity of metabolically sensitive K_ATP_ channels in neurons^24^. In addition, *Bad* knockout mice and liver-specific *Bad* knockdown mice both displayed fasting hyperglycaemia, diminished glycolysis and exaggerated glucose production in the liver^23^, indicating the different functions of *Bad* in the brain and liver. GK rats also had fasting hyperglycaemia and increased hepatic glucose production, but *Bad* was overexpressed in both the hypothalamus (Supplemental Material Figure S2C) and the liver (data from GSE13271^50^). The role of *Bad* overexpression in the GK rat hypothalamus is unclear, is it compensation or exacerbation? The confusing differences between GK rats and other models indicate the complexity of diabetogenic regulation.

The hypothalamus can sense multiple hormones, such as insulin, ghrelin and leptin, and also can sense nutrients, such as glucose. These signals can further trigger activation or inhibition of several hypothalamic neurons, leading to numerous physiological responses by the melanocortin system and the glucose sensing system. We detected the disorder of the melanocortin system and the impairment of the glucose-sensing pathway in GK rats, which may lead to hyperphagia, obstruct signals to glucose-sensitive neurons and further influence the regulation of hepatic glucose production. Several gene network modules, which are enriched for mutations in GK rats, contained various pathways involving hypothalamic function. However, further studies are needed to determine whether and which variants in GK rats influence the regulatory network of the hypothalamus, thus leading to dysfunctional energy homeostasis and the diabetic phenotype of GK rats.

## Conclusion

This study is the first to analyse the hypothalamic transcriptomes at 4, 8, and 12 weeks and population variations in GK rats. The modules using integrated PPI, transcriptomic profile and mutation data were constructed for comprehensive analysis of the GK rat hypothalamus. The GK rat had dysfunction of the hypothalamic melanocortin system and attenuation of the hypothalamic glucose-sensing pathway. The GK-specific genes, such as *Bad*, *Map2k2, Adcy3, Adcy2* and *Gstm6*, may play key roles in hypothalamic regulation in GK rats. Our research provides a comprehensive map of the abnormalities in the GK rat hypothalamus and reveals new mechanisms of pathogenesis in T2D.

## Methods

### Animals and Tissues

GK and Wistar rats (3 weeks old) were purchased from SLAC Laboratory Animal Co., Ltd. (Shanghai, China). Rats were maintained in a P3 room with a 12 hour:12 hour light: dark cycle, a temperature of 20 to 25 °C and 60 ± 5% atmospheric humidity at the Guangdong Key Laboratory of Laboratory Animals. All animals had free access to food and water. All animal manipulations and care were carried out 1.5 to 3.5 hours after the lights were on. This study was approved by the institutional review board of the Guangdong Key Laboratory of Laboratory Animals, and all protocols were carried out in accordance with the approved guidelines of the Institutional Animal Care and Use Committee (IACUC) [Ethic certificate no: IACUC2014029]. Food intakes and body weights of all animals were measured twice each week. Animals were narcotized by pentobarbital sodium (3%, 0.2 ml/100 g) and sacrificed by abdominal aortic exsanguination. Plasma was prepared from the blood with EDTA (4 mM final concentration) by centrifugation (2000 × g, 4 °C, 10 minutes), and stored at −80 °C. Tissues from the hypothalamus were harvested and frozen in liquid nitrogen immediately and finally were transferred into a −80 °C freezer.

### Measurement of plasma glucose and insulin levels

Plasma glucose was measured using a GLU Assay Kit (KOFA, China) using enzymatic methods with an automatic biochemistry analyser (Hitachi 7020, Tokyo, Japan). Plasma insulin was measured by a Luminex MAGPIX using a Milliplex MAG Rat Metabolic Magnetic Bead Panel Kit (insulin kit, Milliplex, Germany).

### RNA extraction, purification and sequencing

In total, 30 hypothalamus samples from 15 GK and 15 Wistar rats at 4, 8 and 12 weeks were prepared for RNA-seq. Total RNA was extracted using TRIzol Reagent (Cat#15596-018, Life Technologies, USA) following the manufacturer’s instructions and the RNA integrity was assessed using RIN (RNA Integrity Number) with an Agilent Bioanalyzer 2100 (Agilent Technologies, USA). Qualified RNA samples were further purified with a RNeasy Micro kit (Cat#74004, Qiagen, Germany) and a RNase-Free DNase Set (Cat#79254, Qiagen, Germany). For RNA-seq analysis, mRNA samples were prepared by using the TruSeq RNA Sample Preparation Kit (Illumina, USA). The mRNA samples were purified first-strand cDNA and second-strand cDNA were synthesized, and the double-stranded DNA underwent end repair. After adenylation of the 3′ ends and ligation of the adapters, PCR was performed to enrich the cDNA templates. Finally, the sequencing library was used for cluster generation (guided by the cBot User Guide) and sequencing on the Illumina HiSeq 2500 system (Illumina, USA) following the HiSeq 2500 User Guide. With the standard paired-end sequencing protocol, 125 bp paired-end raw reads were generated. The kits and equipment used in RNA-seq are listed in Table S4.

### Reads filtering

Low quality reads were filtered using stringent criteria by FASTX (version:0.0.13, http://hannonlab.cshl.edu/fastx_toolkit/): (1) reads with more than 50% of bases with quality <20; (2) the base quality is <10 at the 3′ end of the reads; (3) reads with overrepresented adaptors; (4) reads having an ‘N’ base; (5) reads shorter than 20 bp. After these quality control and filtering steps, clean reads were obtained and used to align the reference mRNA and genome sequences.

### Variant calling

The reads of 15 GK and 15 Wistar rats were mapped to the verified rat reference mRNAs using Bowtie2^51^ with the parameters of -D 20 -R 3 -N 0 -L 20 -i S,1,0.50 -a –no-mixed, which indicates a sensitive end-to-end read alignment programme, outputting with a report of all alignments, and suppressed unpaired alignments for paired reads. The rat mRNA (last updated on November 17, 2014) data were downloaded from NCBI (ftp://ftp.ncbi.nlm.nih.gov/refseq/R_norvegicus/mRNA_Prot/), and the verified mRNAs were kept after filtering the mRNAs with PREDICTED. After filtering by allowing 4 mismatches, 4 gap opens and 4 gap extensions in the alignment and no less than 100 bp alignment reads length, variants were called for each individual by the SAMtools of Version: 0.1.19-44428cd (Tools for alignments in the SAM format)^52^. A variant identical in all 15 GK rats was defined as common GK variants. Similarly, variants unique to the 15 Wistar rats were defined as common Wistar variants. Mutation types of common variants, including coding-synonymous, missense, nonsense and indel, in the coding regions were classified. Then, the genes with common variants present only in GK rats were described as GK-specific genes, and the genes with common variants only in Wistar rats were defined as Wistar-specific genes.

### Differentially expressed genes

Clean data of pair-end reads from each individual were aligned to the *Rattus norvegicus* reference genome (Genome build: rn6) plus transcript indexes by Hisat2 2.0.0-beta^53^, with the parameters of –dta-cufflinks and -sensitive (report alignments tailored specifically for Cufflinks and with sensitive alignment). The mean overall alignment rates were approximately 95% (Table S5). The genome file of *Rattus_norvegicus.Rnor_6.0.dna.toplevel.fa* (ftp://ftp.ensembl.org/pub/release-80/fasta/rattus_norvegicus/dna) and its corresponding annotation file of *Rattus_norvegicus.Rnor_6.0.80.gtf* (ftp://ftp.ensembl.org/pub/release-80/gtf/rattus_norvegicus) were downloaded from the Ensembl database^54^. Cufflinks v2.2.1^55^ was used to process the alignment files and assemble the transcripts. Ballgown^56^, a Bioconductor package, finally estimated the abundance of the assembled transcripts and annotated the genes with FPKM. After these analyses, the gene expression profile with a time-course in rat hypothalamus was determined. Differentially expressed genes between Wistar and GK rats in each period were processed with a Bayes-regularized paired t-test with a FDR correction using Cyber-T bayesreg.R code^57^. For the time-course multiple comparisons, two-way ANOVA tests were applied using TukeyHSD in R.

### Modules

The PPI network was composed of the union of StringDB^58^ v10 rat (organism ID 10116) interactions that were experimentally verified (experimental scores > 400) with high confidence scores (>700), together with the gene-gene relationships of *Rattus norvegicus* in the KEGG pathway database (http://www.kegg.jp/). Four co-expression networks were constructed from the normalized RNA-seq FPKM values at 4, 8, and 12 weeks and all periods. The coefficient r in each pair of genes across all the time points was calculated by R with the Pearson correlation. The modules were detected by integrating PPI, co-expression networks and gene mutations of GK and Wistar rats using MAGI^19^. MAGI first calculated a score for each gene in the networks (by scoring function based on the number of missense and indel mutations and the gene length), selected seed pathways with high scores based on an extension of the color-coding algorithm, and finally improved the modules by adding or removing single genes using a local search^19^. The results of MAGI were contained in *M_Best*, the module that maximizes the score and *M_Extended*, a set of genes that appear in more than 5% of the suboptimal modules (modules with scores within the top 1 percentile that also overlap *M_Best*). To examine whether other distinct modules could be identified, the other genes, which excluded genes in *M_Best*, were used to determine *M2_best* and *M2_Extended* by MAGI. The parameters with MAGI Cluster were as follows: -m 20 -l 35 -u 50 -a 0.6, which indicates the upper bound on control mutations is 20, the lower bound on the size of the module is 35, the upper bound on the size of cluster is 50 and the minimum ratio of seed score allowed is 0.6. The module genes were displayed as graph nodes using Cytoscape^59^, and r^2^ in co-expression networks was set as the edges’ weights. The database for annotation, visualization, and integrated discovery (DAVID) was used to interpret the genes in all modules^60^.

### RT-qPCR verification

Real time quantitative PCR was used to verify the differentially expressed genes using SYBR^®^ Premix Ex Taq™ II (TaKaRa, Japan) with a LightCycler 96 system (Roche, Switzerland). Relative mRNA expression in GK rats was quantified using Wistar rats as the controls and beta-actin (*Actb*) as a housekeeping gene (by the 2^−∆∆Ct^ method). The primers for these genes are listed in Table S6.

### Data availability

The RNA-seq raw data were available in NCBI SRA database (http://www.ncbi.nlm.nih.gov/sra/SRR3923805/).

## Additional Information

**How to cite this article**: Meng, Y. *et al*. RNA-seq analysis of the hypothalamic transcriptome reveals the networks regulating physiopathological progress in the diabetic GK rat. *Sci. Rep.* **6**, 34138; doi: 10.1038/srep34138 (2016).

## Supplementary Material

Supplementary Information

Supplementary Table S1

Supplementary Table S2

Supplementary Table S3

Supplementary Table S4

Supplementary Table S5

Supplementary Table S6

## Figures and Tables

**Table 1:** The details of gene and pathway enrichment in the modules.

	Modules	Gene count	Wistar-snp	GK-snp	Coding-synonymous	Missense	Significantly up-regulated genes	Significantly down-regulated genes	Top10 pathways
**All**	M1	99	25	164	94	70	Bad(1:0;1;0)#; Gstm4(2:0;2;0); Pik3c2b(10:8;2;0); Nt5c3b(1:1;0;0);	Entpd2(0:0;0;0); Itgb4(3:2;1;0); Ppp1r3c(4:3;1;0); Aldh7a1(1:0;1;0); Gstp1(1:1;0;0); Mapk12(0:0;0;0); Alox5(0:0;0;0); Akr1b1(1:0;1;0); Prim1(1:0;1;0);	rno00230:Purine metabolism, rno04914:Progesterone-mediated oocyte maturation, rno04916:Melanogenesis, rno00240:Pyrimidine metabolism, rno04912:GnRH signaling pathway, rno04722:Neurotrophin signaling pathway, rno04910:Insulin signaling pathway, rno04540:Gap junction, rno04270:Vascular smooth muscle contraction, rno04720:Long-term potentiation
	M2	86	23	113	55	58	Gstm4(2:0;2;0); Ifit1(3:1;2;0); Pla2g12a(0:0;0;0); Eif3c(5:4;1;0);	Aldh7a1(1:0;1;0); Gstp1(1:1;0;0); Tdp2(1:0;1;0); Alox5(0:0;0;0); Glb1(0:0;0;0); Tm7sf2(1:0;1;0); Akr1b1(1:0;1;0); Nit2(1:0;1;0); Hmgcs2(1:0;1;0);	rno00980:Metabolism of xenobiotics by cytochrome P450, rno00982:Drug metabolism, rno00480:Glutathione metabolism, rno00650:Butanoate metabolism, rno00590:Arachidonic acid metabolism, rno00830:Retinol metabolism, rno00591:Linoleic acid metabolism, rno04730:Long-term depression, rno00592:alpha-Linolenic acid metabolism, rno00380:Tryptophan metabolism
**4 weeks**	M1	105	28	174	96	78	Bad(1:0;1;0); Ifit1(3:1;2;0);	Entpd2(0:0;0;0); Mrpl44(3:2;1;0); Ppp1r3c(4:3;1;0); Tdp2(1:0;1;0); Mapk12(0:0;0;0); Prim1(1:0;1;0);	rno00230:Purine metabolism, rno00240:Pyrimidine metabolism, rno04722:Neurotrophin signaling pathway, rno04912:GnRH signaling pathway, rno04370:VEGF signaling pathway, rno04910:Insulin signaling pathway, rno04916:Melanogenesis, rno05223:Non-small cell lung cancer, rno04540:Gap junction, rno04012:ErbB signaling pathway
	M2	140	39	229	129	100	Rrp9(9:7;2;0); Eif3c(5:4;1;0); Gstm4(2:0;2;0); Ifit1(3:1;2;0); Pik3c2b(10:8;2;0);	Mrpl44(3:2;1;0); Aldh7a1(1:0;1;0); Tdp2(1:0;1;0); Mapk12(0:0;0;0); Col6a2(7:5;2;0); Gstp1(1:1;0;0); Mlycd(1:0;1;0); Akr1b1(1:0;1;0); Hmgcs2(1:0;1;0);	rno04620:Toll-like receptor signaling pathway, rno05215:Prostate cancer, rno05212:Pancreatic cancer, rno04622:RIG-I-like receptor signaling pathway, rno05222:Small cell lung cancer, rno04914:Progesterone-mediated oocyte maturation, rno05200:Pathways in cancer, rno04920:Adipocytokine signaling pathway, rno04010:MAPK signaling pathway, rno05214:Glioma
**8 weeks**	M1	93	28	180	105	75	Bad(1:0;1;0); Gstm4(2:0;2;0); Pik3c2b(10:8;2;0);	Entpd2(0:0;0;0); Itgb4(3:2;1;0); Ppp1r3c(4:3;1;0); Aldh7a1(1:0;1;0); Mapk12(0:0;0;0); Orc5(0:0;0;0); Dpyd(0:0;0;0); Akr1b1(1:0;1;0); Prim1(1:0;1;0);	rno00230:Purine metabolism, rno00240:Pyrimidine metabolism, rno04916:Melanogenesis, rno04540:Gap junction, rno04914:Progesterone-mediated oocyte maturation, rno04912:GnRH signaling pathway, rno04020:Calcium signaling pathway, rno03030:DNA replication, rno04070:Phosphatidylinositol signaling system, rno04270:Vascular smooth muscle contraction
	M2	144	44	223	136	87	Rrp9(9:7;2;0); Acad9(3:2;1;0); Eif3c(5:4;1;0); Bad(1:0;1;0); Gstm4(2:0;2;0); Ifit1(3:1;2;0); H2afz(0:0;0;0);	Mrpl44(3:2;1;0); Aldh7a1(1:0;1;0); Tdp2(1:0;1;0); Tm7sf2(1:0;1;0); Nit2(1:0;1;0); Pdlim5(2:2;0;0); Ppp1r3c(4:3;1;0); Gstp1(1:1;0;0); Pccb(0:0;0;0); Mlycd(1:0;1;0); Akr1b1(1:0;1;0); Hmgcs2(1:0;1;0); Acat1(1:1;0;0);	rno00620:Pyruvate metabolism, rno00640:Propanoate metabolism, rno00650:Butanoate metabolism, rno04910:Insulin signaling pathway, rno00280:Valine, leucine and isoleucine degradation, rno04620:Toll-like receptor signaling pathway, rno00310:Lysine degradation, rno03010:Ribosome, rno00330:Arginine and proline metabolism, rno00410:beta-Alanine metabolism
**12 weeks**	M1	118	28	190	107	83	Bad(1:0;1;0); Gstm4(2:0;2;0); Ifit1(3:1;2;0); Pik3c2b(10:8;2;0);	Entpd2(0:0;0;0); Mrpl44(3:2;1;0); Itgb4(3:2;1;0); Ppp1r3c(4:3;1;0); Aldh7a1(1:0;1;0); Tdp2(1:0;1;0); Mapk12(0:0;0;0); Mlycd(1:0;1;0); Akr1b1(1:0;1;0); Prim1(1:0;1;0);	rno00230:Purine metabolism, rno04916:Melanogenesis, rno04914:Progesterone-mediated oocyte maturation, rno00240:Pyrimidine metabolism, rno04540:Gap junction, rno04270:Vascular smooth muscle contraction, rno04910:Insulin signaling pathway, rno04730:Long-term depression, rno04912:GnRH signaling pathway, rno04114:Oocyte meiosis
	M2	134	36	202	116	86	Acad9(3:2;1;0); Gstm4(2:0;2;0); Ifit1(3:1;2;0); Pla2g12a(0:0;0;0);	Itgb4(3:2;1;0); Aldh7a1(1:0;1;0); Tdp2(1:0;1;0); Mapk12(0:0;0;0); Prim1(1:0;1;0); RGD735029(2:0;2;0); Vegfb(0:0;0;0); Ppp1r3c(4:3;1;0); Alox5(0:0;0;0); Mlycd(1:0;1;0); Akr1b1(1:0;1;0); Hmgcs2(1:0;1;0);	rno05215:Prostate cancer, rno04370:VEGF signaling pathway, rno04920:Adipocytokine signaling pathway, rno04620:Toll-like receptor signaling pathway, rno05220:Chronic myeloid leukemia, rno05200:Pathways in cancer, rno05222:Small cell lung cancer, rno04730:Long-term depression, rno05212:Pancreatic cancer, rno05221:Acute myeloid leukemia

^#^Gene (mutations: coding-synonymous; missense; indel). For example, Bad has 1 mutation, consists of 0 coding-synonymous, 1 missense and 0 indel.
