# Supplementary material for: RNA-seq analysis of the hypothalamic transcriptome reveals the networks regulating physiopathological progress in the diabetic GK rat
Source: Sci Rep. 2016 Sep 28;6:34138. doi: 10.1038/srep34138 (PMC5039700; doi:10.1038/srep34138)
Supplement: Supplementary Information [file srep34138-s1.doc]

**RNA-seq analysis of the hypothalamic transcriptome reveals the networks regulating physiopathological progress in the diabetic GK rat**

Yuhuan Meng^1,2^, Yujia Guan^1^, Wenlu Zhang^1^, Yu-e Wu^3^, Huanhuan Jia^3^, Yu Zhang^3^, Xiuqing Zhang^2^, Hongli Du^1,*^, Xiaoning Wang^1,4^

1. School of Bioscience & Bioengineering, South China University of Technology, Guangzhou, 510006, China
2. BGI-Shenzhen, Shenzhen, 518031, China.
3. Guangdong Key Laboratory of Laboratory Animals, Guangzhou, 510663, China
4. Chinese PLA General Hospital, Beijing, 100853, China

* [hldu@scut.edu.cn](mailto:hldu@scut.edu.cn)

Table S1. The variants in hypothalamic transcripts.

Table S2. The differentially expressed genes in the hypothalamus.

Table S3. The details of the modules.

Table S4. The RNA-seq equipment and reagent.

Table S5. The information about the sequence reads and genome alignment.

Table S6. The qPCR primers.

Figure S1. Plasma glucose and insulin.

Figure S2. RT-qPCR verification.

Figure S3. The Ct values between the housekeeping gene β-actin (Actb) and the pro-inflammatory factor genes (Il-1b, Il-6 and Tnf) in the RT-qPCR verification.


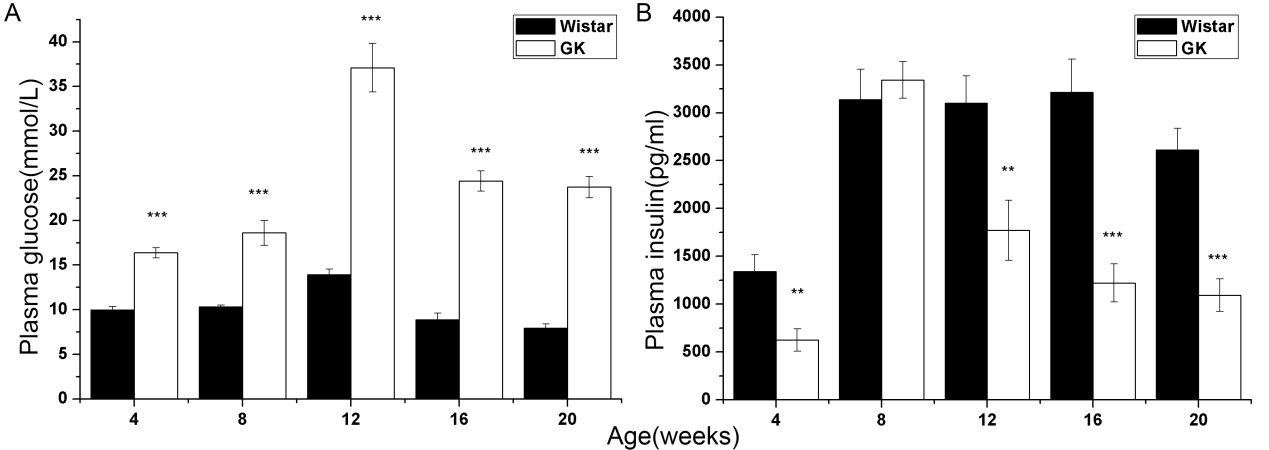


Figure S1. Plasma glucose and insulin(mean ± SE, ***=p<0.01, Student’s t test).


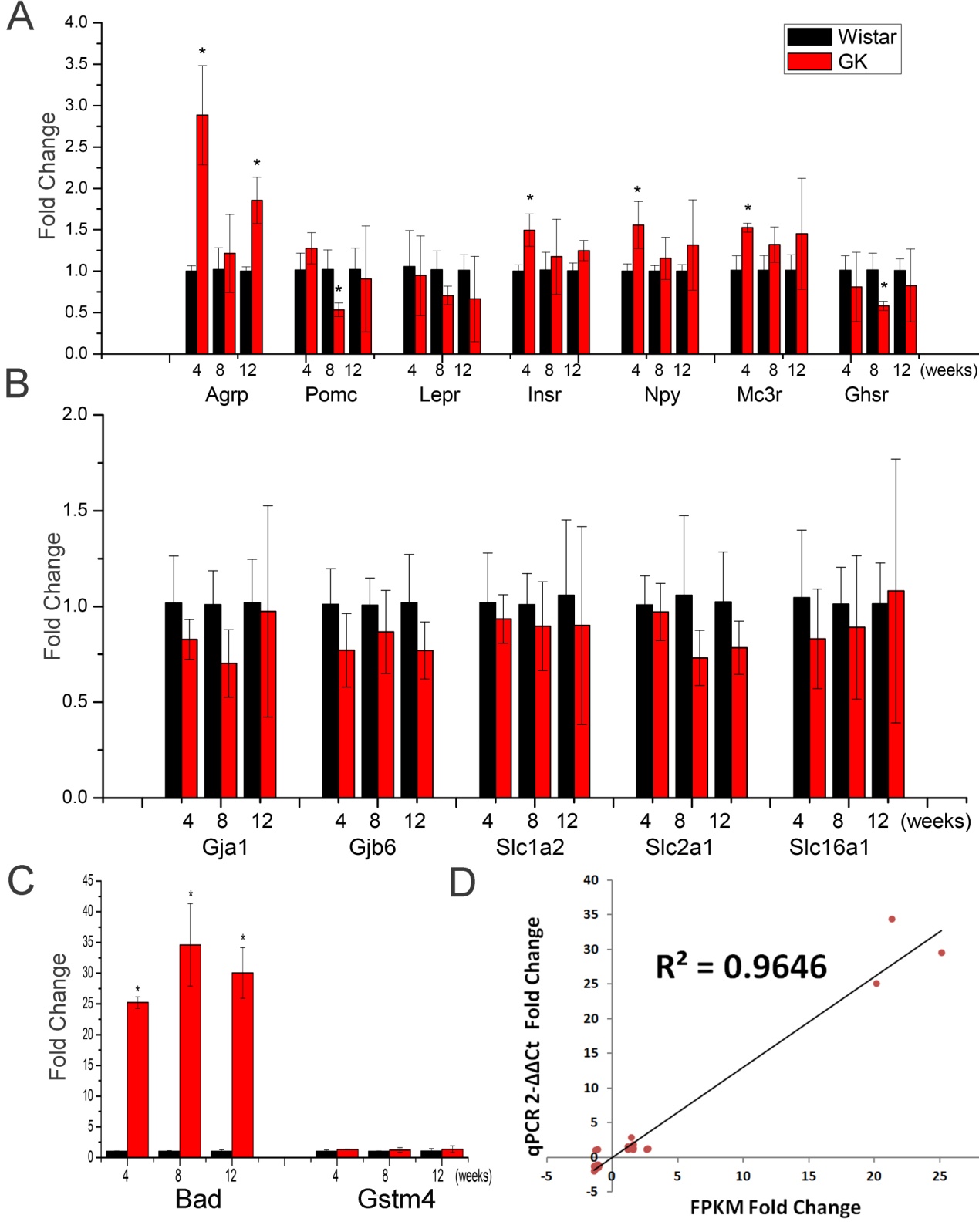


Figure S2. RT-qPCR verification.

1. The verified genes in the hypothalamic melanocortin system;
2. The verified genes in hypothalamic glucose sensing pathway;
3. The verified genes (*Bad and Gstm4*) in modules. Data are shown as the mean ± SD, *, P <0.05 as determined by Student’s t test.
4. The correlation of fold change(GK/Wistar) between RNA-seq and RT-qPCR.


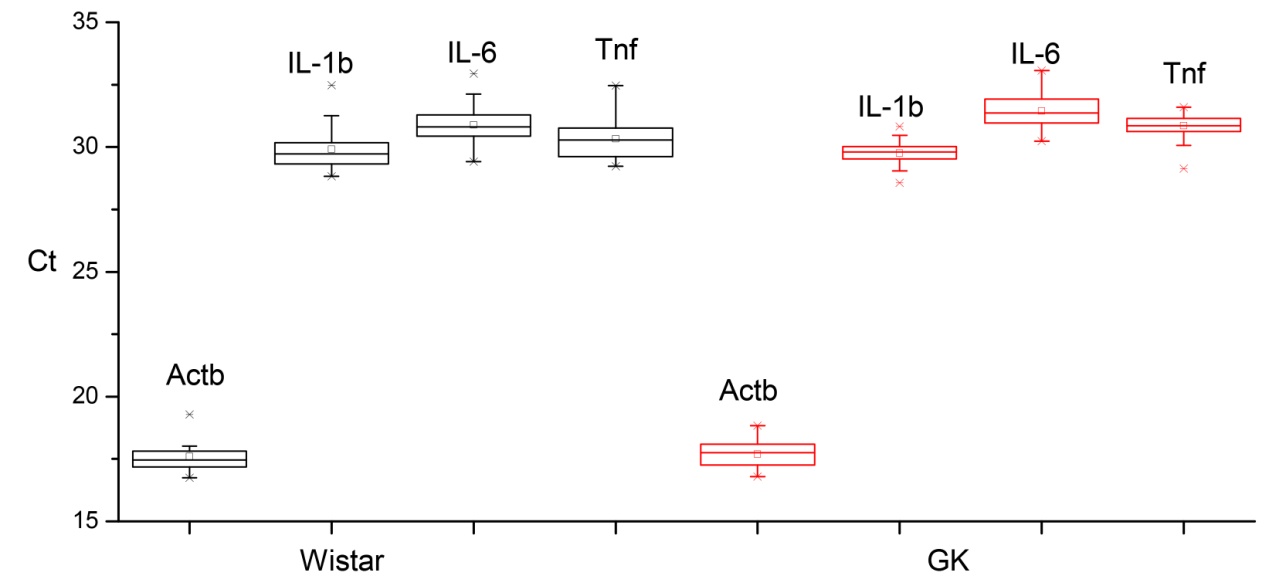


Figure S3. The Ct value between the housekeeping gene β-actin (*Actb*) and pro-inflammatory factor genes (*Il-1b, Il-6* and *Tnf*) in RT-qPCR verification.
